# Multilevel Selection and Neighbourhood Effects from Individual to Metapopulation in a Wild Passerine

**DOI:** 10.1371/journal.pone.0038526

**Published:** 2012-06-20

**Authors:** Paola Laiolo, José Ramón Obeso

**Affiliations:** Research Unit of Biodiversity (CSIC, UO, PA), Oviedo University, Mieres, Spain; University of Manitoba, Canada

## Abstract

Multilevel selection has rarely been studied in the ecological context of animal populations, in which neighbourhood effects range from competition among territorial neighbours to source-sink effects among local populations. By studying a Dupont’s lark *Chersophilus duponti* metapopulation, we analyze neighbourhood effects mediated by song repertoires on fitness components at the individual level (life-span) and population level (growth rate). As a sexual/aggressive signal with strong effects on fitness, birdsong creates an opportunity for group selection via neighbour interactions, but may also have population-wide effects by conveying information on habitat suitability to dispersing individuals. Within populations, we found a disruptive pattern of selection at the individual level and an opposite, stabilizing pattern at the group level. Males singing the most complex songs had the longest life-span, but individuals with the poorest repertoires lived longer than ‘average’ males, a finding that likely reflects two male strategies with respect to fitness and sexual trait expression. Individuals from groups with intermediate repertoires had the longest life-span, likely benefitting from conspecific signalling to attract females up to the detrimental spread of competitive interactions in groups with superior vocal skills. Within the metapopulation selection was directional but again followed opposite patterns at the two levels: Populations had the highest growth rate when inhabiting local patches with complex repertoires surrounded by patches with simple repertoires. Here the song may impact metapopulation dynamics by guiding prospecting individuals towards populations advertising habitat quality. Two fitness components linked to viability were therefore influenced by the properties of the group, and birdsong was the target of selection, contributing to linking social/sexual processes at the local scale with regional population dynamics.

## Introduction

In hierarchically structured systems, natural selection can act at levels higher than the individual, such that individual fitness also depends on the phenotypic expression of neighbours [1]. Context-dependent fitness variation is determined either by multilevel (group) selection or frequency-dependent selection. With group selection, the selective value of a phenotype is a function of the trait expression of social partners, whereas with frequency-dependent selection it depends on the relative phenotypic ranking of individuals within the group [2]. In the latter case, the number of individuals contributing to the next generation in each group is independent of the mean group phenotype, while it is determined by group membership in group selection models [3].

Group selection has been demonstrated in non-kin groups or populations, or in networks of individuals linked by kinship, and in organisms with varied life histories such as animals, plants, fungi, and microbes [4]–[7]. Animals, for instance, experience strong fitness-determining interactions with neighbours in the form of altruistic, despotic or competitive behaviours, which may evolve rapidly when implicated in courtship, reproduction or parental care [8]. On the other hand, frequency dependence explains the maintenance of polymorphisms, clines or alternative strategies resulting from dynamic arms-races within groups [9].

Significant neighbourhood selection can originate at even higher levels than those described above, for instance among local populations within a metapopulation [10]. When there is demographic disequilibrium within a metapopulation, individual performances vary greatly over time, and traits may not experience the same selection in each local population and during different demographic stages of populations [11]. The demographics of the metapopulation in this case create emergent properties that influence the evolution of life history traits (metapopulation selection [12]). For instance, in local populations persisting in a balance between extinction and colonisation, or regularly affected by local extinctions and local overcrowding, population dynamics at each stage may drive the evolution of alternative strategies of dispersal, habitat selection or allocation to reproduction [13]. Empirical evidence is still scarce, but metapopulation selection has been postulated in successional or anthropogenically fragmented environments, in which hard selection can override density dependence and stochasticity in influencing population dynamics and the optimization of life histories [11]. Within metapopulations, selection can eventually feed back to population performance if traits that are advantageous for individuals affect population performance [14]. Life history traits such as dispersal and prospecting behaviour, for instance, may cause mismatches between what is good for the individual and what is good for the population, being costly for the individual but benefitting the persistence of local populations and thus the metapopulation as a whole [15].

Although the spatial organization of metapopulations may engender selection at different levels, applying a multilevel selection framework to ecologically realistic contexts in natural populations is indeed a difficult task [16]. Many phenotypic traits undergoing selection at the individual level may be ecologically irrelevant at the metapopulation scale, and barely affect population performance when resources are limiting or age/sex classes fluctuates strongly, even though traits under selection determine which individuals survive or reproduce [17]. In this context, birdsong offers a unique opportunity to assess under what conditions, and at which level, phenotypic selection on behaviours may have individual and population-wide effects, being a trait mediating reproduction as well as a cue of habitat availability for prospecting individuals in many bird species [18]. As an honest signal of individual quality, birdsong has direct and strong effects on fitness along with other costly sexual ornaments [19]. Moreover, by mediating male-male interactions during breeding, it also causes individual fitness variance via aggressive intrasexual interactions in territorial defence [20] or by eliciting breeding within populations [21]. It therefore creates an opportunity for the action of group selection through the social stimuli of neighbours. In spite of having evolved for a sexual function, the song may guide individual actions in species in which dispersal decisions are based on the perception of cues of the suitability of the surroundings [22]. In birds with advanced sensory abilities, the vocal presence of conspecifics may be such a cue, conditioning colonization rates, rescue effects and the fate of local populations, eventually feeding back to population dynamics [23] and community composition [24].

**Figure 1:**
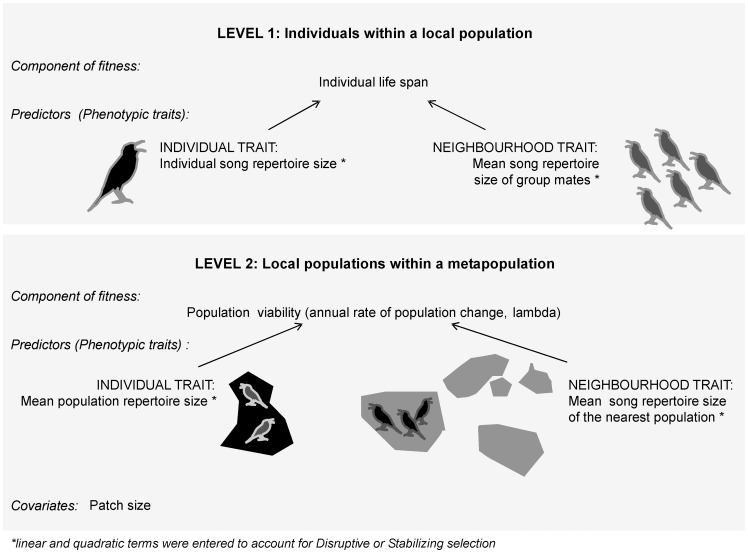
Schematic representation of the units and levels of selection considered in this study.

By studying a system of 19 local populations of a small insectivorous passerine, the Dupont’s lark (*Chersophilus duponti*), we tested for phenotypic selection on song characteristics within local populations and the metapopulation (Figure 1). We first took into account selection within populations, by studying if a component of fitness, life-span, could be predicted by an individual’s and its neighbours’ song repertoire size. We partitioned the relative strength of individual- and group-level selection and tested for directional, stabilizing or disruptive selection on phenotypic traits, and analyzed the interaction between individual and group repertoire size (Level 1 in Figure 1). Sexual signals most often undergo directional selection [25], although disruptive and stabilizing selection may originate from female preference in some cases [26], [27]. In line with studies that showed that repertoire size is an honest signal of male quality and, as such, predicts male survival and reproduction (reviewed in Collins [19]), we expect a directional positive selection on large repertoire sizes, also taking into account the positive effect that large repertoires have on the productivity of Dupont’s lark populations [28]. We incorporated the group character into models (neighbours’ song), expecting that group song complexity negatively influences individual performances via interference competition among Dupont’s lark males, which engage in countersinging disputes during dawn choruses [29].

In a higher order analysis at the metapopulation level, we considered population song repertoire as a determinant of population viability (the annual rate of population change, λ), after controlling for environmental variation within the metapopulation (Level 2 in Figure 1). The song repertoire of the population and that of the nearest populations were considered as within- and among-population traits, since we expected that conspecific attraction mediated by the song could regulate metapopulation dynamics, increasing dispersal towards populations singing attractive (complex) songs [28].

## Materials and Methods

### Ethics Statement

The study was performed under proper legislation of the Spanish law. The methods we have used (song recording) were not subject to restrictions in our study area, and did not cause any nest desertion or mortality or stress to birds.

### Study System

We made use of information on demographic parameters of a Dupont’s lark metapopulation that was systematically surveyed from 2004 to 2008 in Ebro Valley, NW Spain [29]–[31] (details in Appendices S1, S2, S3, S4). The Ebro Valley is the second largest stronghold of Dupont’s lark in Spain but its habitat is highly threatened by anthropogenic activities; the overall number of occupied territories is estimated as *ca*. 680, split into small local populations holding from 2 to 50 territories [32] Steppe patches cover from 5 to 800 ha and are located at 2–24 km from each other [31], [32] (Appendix S1). Inter-patch movements by males are uncommon (3% of marked individuals) and, when they do occur, limited to <20 km from the natal population [32]. Dupont’s lark populations interact in a source-sink dynamic in which small, isolated or marginal nuclei have the greatest probability of extinction [32]. In historical times, gene flow has been reduced by anthropogenic land transformation, and drift is most intense in the smaller and more isolated populations [33]. For this study we focused on 19 of these populations to partition the effects of individual and neighbour phenotypes on fitness (individual and population levels), and the effects of local population and neighbour population phenotypes on viability (population and metapopulation level). During the study period, the demographic factors that mainly affect song repertoire variation in the Dupont’s lark had not changed (population size and territorial displacements) [30], and thus we can reasonably assume that the social milieu was stable throughout the 5 years of this study.

### Phenotypic Trait: Song Repertoire

Reviews of birdsong function and evolution across a range of studies indicate that the complexity of song elements (repertoire size) is an indicator of male reproductive effort [34] and cognitive ability [35], and may correlate with reproductive success [36], [37]. In the Dupont’s lark, males signal aggressiveness to neighbours by countersinging at territory borders during the dawn chorus in spring, and individuals confront each other by copying their repertoires (Appendix S2) [29]. Singing activity also affects immigration since dispersal most often occurs towards actively vocal local populations of this lark [23], and the song complexity indicates patch quality to dispersing individuals [28]. Population productivity correlates significantly with individual song repertoires, with complex songs being associated with high reproductive success [28]. Moreover, the repertoire differs among local populations, but is less sensitive than other acoustic parameters, such as spectral and temporal patterns, to the influence of bird morphology or habitat structure [38].

The song repertoire of the Dupont’s lark was quantified as the number of discrete strophes in an individual song (Appendix S2). Although this is often a reliable indicator of bird condition in passerines [19], it does not appear to be age or experience-dependent in the Dupont’s lark: the repertoire is maintained unchanged throughout life, which is relatively short (average life expectancy  = 1.7 years ±0.07 SE, *n* = 160 males). Similar to other short-lived species with early close-ended learning, the song repertoire may reflect fixed aspects of male quality (genotype) and/or life-long consequences of bird social or environmental conditions early in life [35], [39]. Songs for this study were recorded over four years only (2004–2007), a period in which population repertoires did not change [28].

For this study, the individual repertoire size was the individual phenotypic trait, and the average repertoire size of an individual’s group mates (excluding the focal individual) was the neighbour phenotypic trait in multilevel selection analyses at the population level (Figure 1). At the metapopulation level, the average repertoire size of population members was the population phenotypic trait, and the average repertoire size of the members of the nearest neighbour population was the neighbourhood phenotypic trait (Figure 1). In the first approach, we considered several neighbours as group mates, since males may engage in territorial dispute with several neighbours at a time during dawn choruses (Appendix S2) [29]. Nevertheless, in the latter we considered the closest population, located up to 23 km distance, in order to focus on interacting populations only (e.g. those potentially involved in the reciprocal exchanges of individuals [32]).

### Components of Individual and Population Performances: Life-span and the Annual rate of Population Change

The number of descendents per reproductive season and survival are the two major components of fitness (intended as Fisher’s reproductive value [40]). Although they may be linked by constraining relationships, survival is less commonly affected by trade-offs than other life-history traits (vegetative growth, adult fecundity, future reproduction; reviewed by Linden & Møller [41] and Obeso [42]). In vertebrates, survival or life-span are often used as surrogates of fitness (“viability fitness”) when studying the evolution of quantitative traits [birds: Clegg *et al.* [43]; reptiles: Janzen *et al.* [44]; fishes: Dibattista *et al.* [45]]. This is because descendants in wild living populations may emigrate from their populations and their reproductive value may be difficult to track, social and genetic fathers can be confounded, and the effects of mate quality and investment may be difficult to parse out [39]. Therefore, survival better approximates life-time reproductive success and can be estimated with lower uncertainty than other life-history traits, such as seasonal fecundity or the overall number of descendents [41].

In small passerines, age-specific changes in survival and reproductive success often run in parallel [46], [47], and in conditions of high environmental variability longer life-spans guarantee reproducing at least once [48]. The low productivity of typical lark habitats and the high nest predation rates render reproductive failures frequent in this group, to the extent that some individuals may forego breeding entirely in bad seasons [49], [50]. In these conditions, the parental effort and the fitness value of single broods is reduced [51], and fitness is strongly predicted by survival or life-span. In the Dupont’s lark, the fitness surrogate that could be quantified with the lowest uncertainty was life-span, which was established by monitoring populations with the acoustic-marking technique. The rationale of this marking method, developed for studying elusive or capture-sensitive species, is that recording a calling individual (and defining the acoustic characteristics of its call) is equivalent to physical marking, such that recording that male in successive sessions is comparable to recapture [52], [53]. The territorial call of Dupont’s lark is individually distinctive and constant over time, thus permitting us to track individual movements and estimate the life-span of the caller (total years from first appearance with a fully developed call to disappearance). Recordings were obtained in spring and summer-autumn in 2004–2008. During each season and year, transects were repeated in each steppe patch of the Ebro Valley, and calling birds were approached (<30 m) to obtain good quality recordings. Details on the acoustic marking technique, as summarized from published information, are provided in Appendix S3.

The group’s probability of persisting (fitness) was expressed here by the relative rate of population growth (or annual rate of population change, λ), an emergent property of the population that expresses the probability of surviving given environmental stochasticity. This parameter was obtained by means of population viability analyses (PVA), as detailed in Laiolo *et al.* [28], Vögeli *et al.* [31] and Appendix S4. The annual rate of population change (λ) summarizes variation in other surrogates of population performance (population size and productivity) and covaries with other population viability indices, such as the median extinction time, the mean extinction time and the exponential rate of increase [28], and was chosen as a proxy of fitness at the population level. Since it was estimated independently from the component of individual fitness (life-span), it does not represent a higher-level statistic analogous to the latter.

At the lower level of analysis, we calculated the relative fitness by dividing individual life-span by mean group life-span (excluding the focal individual). To estimate the individual and group trait, we considered the song repertoire of 32 singing males of known life-span (inhabiting 15 populations) and that of their neighbours (totalling 116 singing males from 15 populations), respectively. In metapopulation level analyses, the relative group fitness was calculated by dividing the population λ by the mean λ of all populations (excluding the focal one). Our sample is based on 19 populations of known-viability and song traits (of the focal population and of the nearest one, for a total of 155 singing males).

### Data Analysis

Before testing for phenotypic selection, we explored the influence of the environmental, geographic and demographic context on absolute fitness at different levels, in order to quantify the magnitude of their impact and to parse out confounding spurious covariations [5]. By means of multiple regressions we tested for the effects of population size and geographic location (coordinates) on fitness components, because of the broad geographic and demographic gradient embraced by this study, which may hinder local trends. Moreover, we considered the size of the habitat patch and its primary productivity (monthly maximum value of the satellite-derived NDVI) as indicators of habitat quality (estimates in Laiolo & Tella [54]; Vögeli *et al.* [32]).

#### Level 1: Individual and population level selection

We partitioned the effects of individual and population phenotypes on relative individual fitness by means of generalized linear mixed models GLMM with a Gaussian distribution of errors, adding population identity as a random factor to control for the possible non-independence of individual parameters within populations. We tested for a model including only linear terms, assuming that the song repertoire size of the individual and that of its neighbours linearly affect individual fitness (i.e. selection is directional). We also tested for a model entering quadratic terms, to account for non-linear phenotype-fitness trends produced by disruptive and stabilizing selection [55]. In both models, we also entered an interaction term between individual and neighbour traits. The strength of selection directly on the characters was estimated by running multiple regression models to generate partial regression coefficients, either linear (β) or quadratic (γ) (Appendix S5), as well as their significance [55], [56]. The curvature of the phenotype-fitness trends permits us to distinguish between stabilizing and disruptive selection on traits: in the former case quadratic selection gradients are negative (γ<0), whereas in the latter they are positive (γ>0).

#### Level 2: Population and metapopulation level selection

We tested for linear and quadratic effects of mean population trait and mean neighbour-population trait on population relative λ by means of multiple regression models (Appendix S5).

To exclude any effect of sexual signal variation on fitness, we tested for the correlation between relative life-span and the coefficient of variation of neighbour repertoire size, and between population relative λ and the coefficients of variation of the repertoire size the population and of the nearest population. We found no significant effects either on life-span (*t* = 0.51, *P* = 0.61, n = 32 individuals) or λ (all *t*<0.88, *P*>0.39, n = 19 populations). Statistical analyses were performed with R [57] and Statistica 6.0.

## Results

### Fitness Relationships with the Environment

Individual absolute life-span was not significantly affected by group size (*R* ^2^ = 0.035; *F*_1,30_ = 0.011, *P* = 0.74), geographic coordinates (*R* ^2^ = 0.018, *F*_2,29_ = 0.26, *P* = 0.77), patch size (*R* ^2^ = 0.005, *F*_1,30_ = 0.13, *P* = 0.71) or primary productivity (*R* ^2^ = 0.004, *F*_1,30_ = 0.12, *P* = 0.72). Patch size was the only environmental variable that significantly (and positively) affected population absolute λ (β = 0.52±0.21, *R* ^2^ = 0.27, *F*_1,17_ = 6.3, *P* = 0.022), and was therefore entered as a covariate in multiple regression models of metapopulation level analyses; geographic coordinates, group size and plant productivity had no significant effect (all *R* ^2^<0.16, *F*<3,2, *P*>0.09).

### Level 1: Individuals within the Population

**Figure 2:**
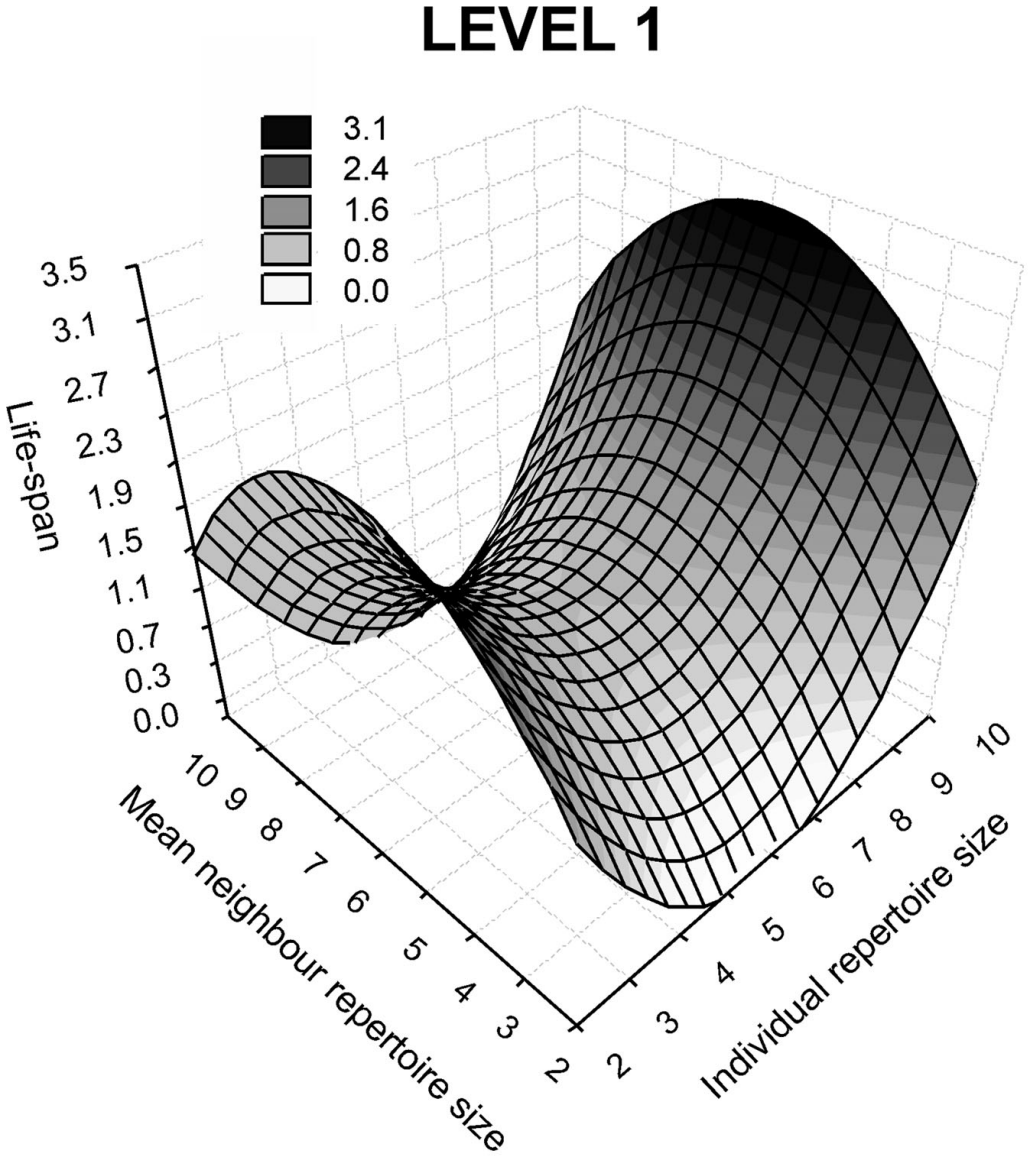
Three-dimensional fitness surface with increasing values of life-span expressed in a grey scale.

GLMMs controlling for population identity showed non-significant effects of both individual and neighbour repertoire size on relative life-span when they were entered as linear terms (*t* = 0.74 and 1.30, *P* = 0.46 and 0.20, respectively), as well as non-significant effects of the interaction between individual and neighbour repertoire size (*t* = 1.22, *P* = 0.23). On the contrary, the model entering the linear and the quadratic term of individual and neighbour traits showed significant effects of both variables (individual repertoire: *t* = −3.9, *P* = 0.0018; square individual repertoire: *t* = 4.1, *P* = 0.0012; neighbour repertoire: *t* = 2.6, *P* = 0.020; square neighbour repertoire: *t* = −2.7, *P* = 0.018). Contextual analysis confirmed that phenotypic selection significantly deviated from linearity with respect to both individual and neighbour traits and fitted quadratic models with opposite curvatures (Table 1 A shows the results of multiple regression models; when quadratic terms were excluded tests were not significant, individual β = 0.18, *F*_1,29_ = 0.31, *P* = 0.58; group β = −0.14, *F*_1,29_ = 0.19, *P* = 0.66). The result is a stabilizing pattern of variation concerning the neighbour trait and a disruptive pattern for the individual trait, with those individuals singing simple or the most complex repertoires having the highest relative life-span, and populations with intermediate repertoires hosting relatively longer-lived males than smaller or larger repertoire-size groups (Figure 2). Males singing complex songs were the fittest in populations with rich repertoires, but the least fit in poor-song populations (Appendix S6). The disruptive pattern was not symmetrical (Figure 2 and Appendix S7), and Dupont’s lark males with the most complex songs outperformed individuals with the simplest songs.

**Table 1:** Results of contextual analysis performed on individuals of known life-span and song repertoire size (A; *n* = 32 individuals), and on populations of known annual rate of population change λ and song repertoire size (B, *n* = 19 populations).

A) Level 1: Effects on life-span	Selection gradients (SE)	Type of selection
Individual repertoire size	β = − 0.60 (0.153)***	
(Individual repertoire size)^2^	γ = 1.24 (0.151)***	Disruptive
Mean neighbour repertoire size	β = 0.45 (0.172)*	
(Mean neighbour repertoire size)^2^	γ = − 0.92 (0.171)*	Stabilizing
B) Level 2: Effects on λ		
Mean population repertoire size	β = 0.58 (0.18)**	Directional
Mean repertoire size of the nearest neighbour population	β = − 0.39 (0.16)*	Directional
Patch size	β = 0.32 (0.19)	

Patch size was the only covariate significantly correlated with λ when entered alone, and was therefore entered in model at Level 2. * P<0.05, ** P<0.01, *** P<0.001.

### Level 2: Populations within the Metapopulation

**Figure 3:**
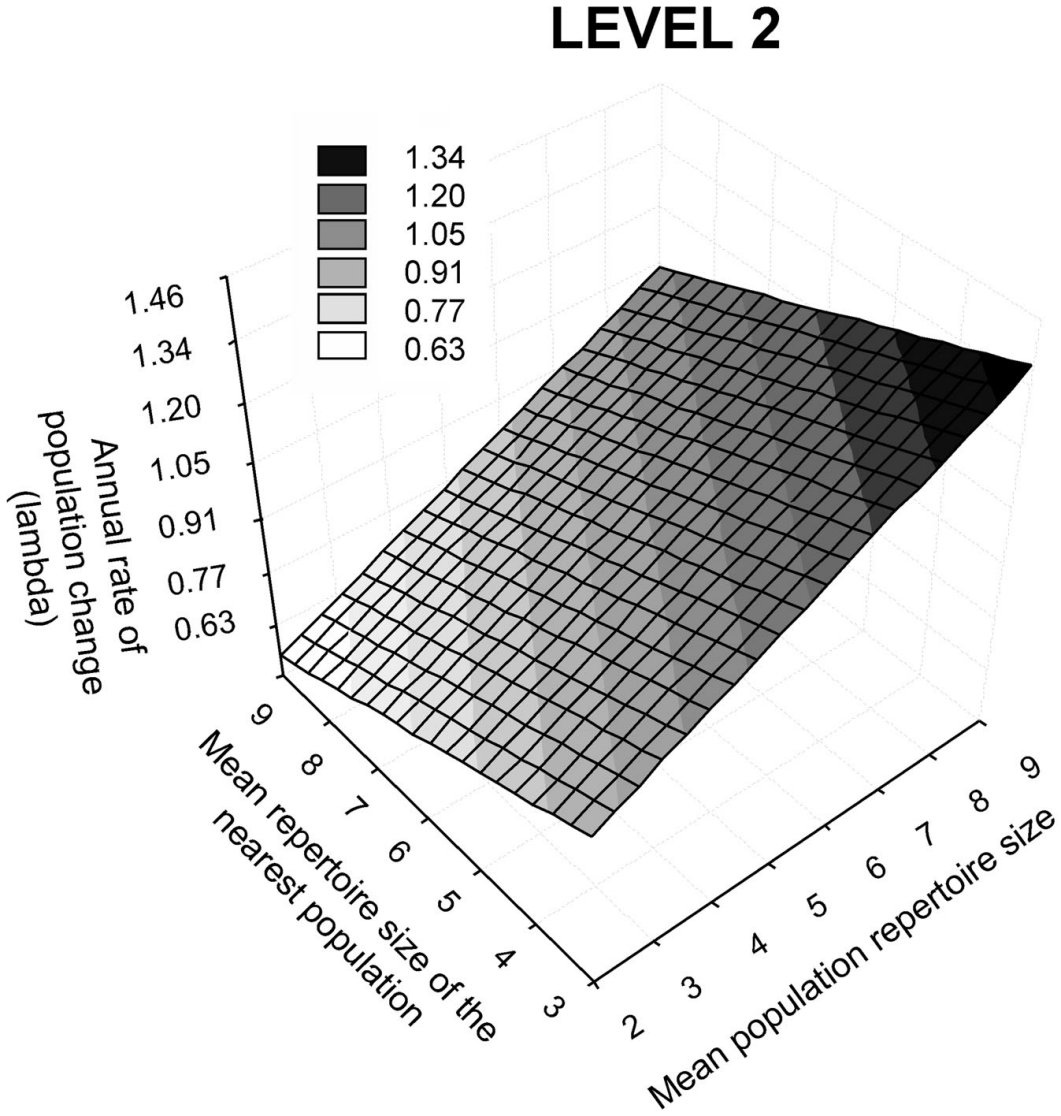
Three-dimensional fitness surface with increasing values of the annual rate of population change expressed in a grey scale.

The repertoire of the neighbour populations had no significant effect on individual life-span, implying that metapopulation selection did not influence this component of individual fitness (β = −0.009, *F*_1,30_ = 0.003, *P* = 0.96). However, it affected group fitness: λ significantly increased in populations characterized by complex songs (*t* = 3.11, *P* = 0.007) and in populations located close to populations singing simple songs (*t* = 2.40, *P*<0.030) (Table 1 B; Figure 3; Appendix S8). The annual rate of population change was not affected by patch size when this variable was entered in the above model together with the other variables (*t* = 1.64, *P* = 0.12; Table 1 B). The quadratic and interaction terms of population and neighbour-population repertoire size were not significantly different from zero (all *t*<1.5, *P*>0.16), nor did the size of the nearest patch have an effect (*t* = 0.61, *P* = 0.54).

## Discussion

Although both empirical studies and theoretical models have shown that group selection can contribute to evolutionary responses [2], [58], [59], neighbourhood effects and their population-level consequences are still poorly documented in the wild, especially in the context of metapopulations [16]. Evidence is almost nil for wild vertebrates, in spite of the fact that the wide application of capture-recapture models has permitted estimates of (viability) selection otherwise difficult to obtain in free living populations [60]. Our study on the communication behaviour of a wild passerine shows that two fitness components linked with viability-the life-span of individuals and the annual rate of population change -are influenced by the properties of the group, represented by territorial neighbours at the individual level and by the nearest neighbouring local population at the metapopulation level, as detailed below.

### Level 1: Individual Disruptive Selection

Sexual selection theory predicts that longevity can covary either positively or negatively with sexual signal expression: Pure Fisherian models expect a negative correlation, in line with life-history theory and resource partitioning between attractiveness and survival, whereas honest signal theory predicts a positive covariation [61]. Both cases are empirically supported by song repertoire studies [39], [62]. Between these extremes, the interaction of selection forces may also lead to non-linear effects [63], a phenomenon that is apparently emerging in the Dupont’s lark, where signal-fitness covariation shows a strong disruptive pattern smoothed by the group effect (Figure 2). Individuals with the largest song repertoires appear to be of the highest quality, achieving the greatest survival in competitive populations (i.e., where males sing complex songs), but those with the simplest repertoire outperform ‘average’ males, especially when surrounded by simple-song neighbours. This pattern suggests that low-cost mating strategies may have evolved to increase fitness while reducing signalling. Marshall et al. [64] found that social fathers sang complex repertoires but genetic fathers (extra-pair mates) sang simple songs in the sedge warbler (*Acrocepalus schoenobaenus*), demonstrating that females use different cues to choose mates-the song repertoire as an indicator of paternal offspring provisioning and/or territory size, and other features from an extra-pair mate. Alternative strategies are often adopted by young or by floaters, e.g. the section of the male population that cannot pay the costs of territorial maintenance and advertisement. The number of floaters is indeed high in the Dupont’s lark [29], and cuckoldry associated with extra-pair paternity has been described in several lark species [65], [66]. Both phenomena can drive the evolution of alternative mating strategies, and by adopting them some Dupont’s lark individuals may perform better while reducing signal complexity. Although there is scarce evidence of disruptive selection on sexual signals in birds, the phenomenon has been described in vertebrate species with alternative strategies (or forms) that persist through negative frequency-dependent female choice, such as in cichlid fishes [67] or lizards [68].

Estimates of selection gradients (partial regression coefficients β and γ) across literature generally indicate weak selection for morphological traits and a stronger selection (e.g. faster evolution) for traits involved in mating [69]. We acknowledge that our estimates (Table 1 A) may be biased upwards by a low sample size, or by the covariance between phenotype and some environmental factor we were unable to detect. However, these high β- and γ-values are in line with the idea that characters that directly feed-back to mating, such as ornaments that clearly imply an expectation of mate choice, may be the objects of the strongest selection regimes [69].

### Level 1: Group Stabilizing Selection

Dupont’s larks surrounded by neighbours with intermediate repertoire size perform significantly better than males from rich- or poor-song groups (Figure 2). The intermediate conditions generated by average-signalling neighbours may maintain an optimal balance between two opposing social forces within groups: facilitation, guaranteeing attractiveness for females, and male-male competition for territories and mates. These forces may interact in a dynamic equilibrium that starts with an initial amelioration of the chance of success of individuals as neighbour signals improve, up to the spread of competitive interactions between the initial passive (i.e. unwilling) facilitators and their beneficiary. This symmetric equilibrium appears to benefit individuals expressing a wider range of individual repertoires, at the same time softening the strong disruptive pattern found at the individual level.

Social facilitation, when individual performance benefits from the signalling of conspecifics, has been demonstrated in animal-pollinated plants [70], frogs [71], and birds [21], [72]. In the Dupont’s lark, attraction for signalling conspecifics may lead dispersing females to settle preferentially where males sing attractive (i.e. complex) songs [23], [28] (see also below). In groups with complex songs, on the other hand, antagonistic interactions among males can outweigh facilitation benefits of social signals [24]. In the Dupont’s lark, song sharing and matching, i.e. the copying of neighbours’ song strophes, serves to defend territories from rivals. This strategy may render the interaction with males with superior vocal skills a demanding activity [29].

All in all, in the Dupont’s lark disruptive selection at the individual level is likely associated with two alternative male strategies with respect to fitness. These may originate from females seeking different qualities from a pair mate than from an extra-pair mate, and a possible explanation for this behaviour is that it increases the genetic variety within the clutch, with offspring adopting both strategies of their parents [73]. At the group level, phenotypic selection is acting in the opposing direction, following a stabilizing pattern that is uncommon for the most popular multilevel settings in plants and animals (self-thinning and altruism, respectively) [1]. Group selection in the Dupont’s lark is likely indicating underlying correlational selection on traits that become overt through sexual signal expression, such as aggressiveness and attractiveness. The strength of the evolutionary response to this phenotypic selection depends on the relative magnitude of the genetic *versus* epigenetic (cultural) component of the trait considered.

### Level 2: Neighbourhood Effects on the Annual Rate of Population Change λ

At the broader scale of the metapopulation, λ also varies with respect to the phenotype of the population and of the neighbour population. However, selection is directional at both levels, and λ increases in populations with complex songs surrounded by patches with simple songs (Figure 3). It has been suggested that selection on dispersal-associated behaviours (sampling of the environment and access to information) may affect population performance and metapopulation dynamics via an impact on migration rate [15]. In the Dupont’s lark, dispersal and prospecting behaviours may be involved in these metapopulation level effects, since these traits influence the connectivity and performance of populations in conditions of anthropogenic habitat shortage. Social attraction phenomena have been documented in the species, and the fixation of behaviours that help in ‘choosing’ proper neighbours may have evolved to maximize the fitness of dispersing inexperienced (young) individuals [23]. Population-level effects may result either from similar dispersal strategies of females and males, or from females only assessing environmental quality by cueing on male ornaments [22]. Although we are unable to separate these two components at the present level of knowledge, we can argue that attractive signalling may function as a cue of habitat and mate quality chiefly in the case of females, since mates with the most complex songs in the Dupont’s lark survive longer and likely perform better than males with average or poor repertoires. This behaviour also helps to explain the highest productivity of the fragments inhabited by males with complex songs [28]. Males, on the other hand, may face survival drawbacks in highly competitive environments since group selection favours individuals surrounded by intermediate phenotypes. Although there is no evidence in the Dupont’s lark, it has been shown in other passerines that dispersers may perceive competition levels *via* song stimuli, and consequently avoid the costs of competition by selecting patches with intermediate signal intensity [24].

When comparing individual and population fitness, therefore, it appears that survival benefits for males are gained when settling among neighbours that are neither too competitive (complex song) nor poorly competitive (simple song). On the other hand, certain individuals (likely females) would benefit from migrating to a neighbour population with complex songs if males from local populations poorly advertise their qualities, eventually increasing the chance of persistence of the nearest population because of their immigration.

Although our discussion is speculative in several aspects, this study aims to stimulate the interest in the variety of social selection pressures that can propel evolutionary changes that eventually feed back to population dynamics [15]. In this context, animal populations offer a wide set of models from solitary to highly complex social systems (breeding colonies, aggregations of foraging and roosting individuals, etc.) in which the individual and group contribution to life-history evolution and population dynamics could be disentangled with a multilevel selection approach [8].

## Supporting Information

Appendix S1 Location of the study area and of the 19 populations of the Dupont’s larks recorded in Ebro Valley. The mean song repertoire of each male population, the number of males recorded singing and population size (expressed as the number of occupied male territories) are provided in a table.
(DOC)

Appendix S2 Analysis of song repertoire size.
(DOC)

Appendix S3 Acoustic marking technique.
(DOC)

Appendix S4 Group fitness components: population viability.
(DOC)

Appendix S5 Contextual analysis models and parameters.
(DOC)

Appendix S6 Relationship between relative life-span and individual repertoire size as observed in 32 Dupont’s lark males. Individuals are classified as belonging to groups with average, large (> average+1SE) and small (< average−1SE) repertoires. Trend lines are also shown.
(DOC)

Appendix S7 Relative life-span of Dupont’s lark males as predicted by their repertoire size and that of their group mates by the multiple regression model. Multiple regression coefficients obtained from 32 birds of known life-span were fitted to the songs of 155 males from 19 groups.
(DOC)

Appendix S8 Relationship between relative population viability (λ) and the mean song repertoire size of the local population (A) and of the nearest population (B) as observed in 19 populations of the Dupont’s lark. Residuals of the regression of λ on mean population repertoire were used instead of raw values in plot B.
(DOC)
