# Supplementary material for: Multilevel Selection and Neighbourhood Effects from Individual to Metapopulation in a Wild Passerine
Source: PLoS One. 2012 Jun 20;7(6):e38526. doi: 10.1371/journal.pone.0038526 (PMC3380010; doi:10.1371/journal.pone.0038526)
Supplement: Appendix S1 — Location of the study area and of the 19 populations of the Dupont’s larks recorded in Ebro Valley. The mean song repertoire of each male population, the number of males recorded singing and population size (expressed as the number of occupied male territories) are provided in a table. (DOC) [file pone.0038526.s001.doc]

**Appendix S1.** Location of the study area and of the 19 populations of the Dupont’s larks recorded in Ebro Valley. The mean song repertoire of each male population, the number of males recorded singing and population size (expressed as the number of occupied male territories) are provided in the table.
