# Supplementary material for: Multilevel Selection and Neighbourhood Effects from Individual to Metapopulation in a Wild Passerine
Source: PLoS One. 2012 Jun 20;7(6):e38526. doi: 10.1371/journal.pone.0038526 (PMC3380010; doi:10.1371/journal.pone.0038526)
Supplement: Appendix S2 — Analysis of song repertoire size. (DOC) [file pone.0038526.s002.doc]

**Appendix S2. Analysis of song repertoire size.**

In March-June 2004-2007 we recorded Dupont’s lark males in their territories, from 1-1.5 hr before until 1-2 hr after dawn. We used a TC-D8 DAT recorder (Sony Corporation, Japan) and a ME67 microphone (Sennheiser, Germany) to record birds, and a GPS eTrex® Navigator (Garmin, USA) to establish male positions during singing bouts. During dawn choruses, males from neighbouring territories often approach each other to countersing along territory boundaries, as shown below.

A Dupont’s lark song unit is made up of several discrete sequences (2 -12 per individual); song sequences (types) are made up of 1-13 syllables. Sound analyses were carried out with Avisoft SASLab Pro 3.91 (Specht 2003), performing a Fast Fourier Transform (sampling frequency 22050 Hz, FFT length 512, time resolution 8.9 msec, frequency resolution 43 Hz, Window Function: Bartlett). To quantify individual repertoire size, e.g. the number of different song types in an individual repertoire, we classified song types by inspecting sonograms by eye. Visual inspection of sonograms was carried out by the same investigator, thus avoiding any interobserver bias. Details on Dupont’s lark behaviour while singing, and on spatial variation in Dupont’s lark song repertoires can be found in Laiolo and Tella (2005, 2007 a, 2007b), Laiolo et al. (2008) and Laiolo (2008).

**References**

Laiolo, P. & Tella, J.L. (2005) Habitat fragmentation affects culture transmission: patterns of song matching in Dupont’s lark. *Journal of Applied Ecology* **42**, 1183-1193.

Laiolo, P. & Tella, J.L. (2007)a. Erosion of animal cultures in fragmented landscapes. *Frontiers in Ecology and the Environment* **5**, 68-72.

Laiolo, P. & Tella, J.L. (2007)b. Vocal diversity patterns - Reply. *Frontiers in Ecology and the Environment* **5**, 406-407.

Laiolo, P., Vögeli, M., Serrano, D., & Tella, J.L. (2008). Song diversity predicts the viability of fragmented bird populations. *PLoS-ONE* **3**, e1822

Laiolo, P. (2008). Characterizing the spatial structure of songbird cultures. *Ecological Applications* **18**, 1774-1780.

Specht, R. (2003) Avisoft-SASLabPro. Sound Analysis and Synthesis Laboratory, version 4.23e. Avisoft Bioacoustics, Berlin (Germany).
