# Supplementary material for: Multilevel Selection and Neighbourhood Effects from Individual to Metapopulation in a Wild Passerine
Source: PLoS One. 2012 Jun 20;7(6):e38526. doi: 10.1371/journal.pone.0038526 (PMC3380010; doi:10.1371/journal.pone.0038526)
Supplement: Appendix S3 — Acoustic marking technique. (DOC) [file pone.0038526.s003.doc]

**Appendix S3. Acoustic marking technique.**

Here we provide details on the acoustic marking technique, as summarized from published information (Laiolo & Tella 2006, Laiolo et al. 2007, Vögeli et al. 2008, Laiolo et al. 2008). To demonstrate that territorial calls can be used for individual identification (i.e. that vocal features remain constant over time), recordings of 36 banded birds in the period 2004-2005 were analyzed. On different days, playback sessions were repeated during daylight to attract, identify and record the vocal response of territorial birds. This technique allows the simultaneous recording and identification of colour-ringed birds, which are otherwise difficult to observe while calling at night or hidden in the vegetation. Sound analyses were carried out with Avisoft SASLab Pro (Specht 2003); acoustic characteristics were measured in the time domain on envelopes and in frequency domain on fast Fourier transform mean spectra. In each call type (10 overall in the Ebro Valley), we measured overall call duration, the duration and the frequency of each note; when partly or entirely frequency-modulated (i.e. when frequency changed over time) or harsh notes occurred, we also measured the duration, the maximum and minimum frequency of the modulated or harsh portion, and the number of modulations. The number of spectrotemporal variables measured varied from 4 in the simplest call type (1 syllable) to 17 in the longest call (5 syllables) (see Laiolo et al. 2007 for spectrograms and details on measurements). A matrix was built in which individual identity was listed in rows, and the acoustic variables of all the call types in a train were arranged in columns. Discriminant function analysis was used to test for the discriminant power of the acoustic features of these 36 birds of known identity. To measure similarity between territorial calls we also calculated the Euclidean distances between the acoustic features of pairs of birds (dyads). From the pairwise matrix, we calculated acoustic distances for dyads of known identity: (a) same bird-same year, (b) same bird-different year, (c) different bird-same territory, (d) different bird-same steppe patch, and (e) different bird-different steppe patch. Discriminant function analysis correctly classified 100% of call trains to the individual from which they were recorded, either recorded within the same day, in different days, seasons or years (Laiolo et al. 2007). The maximum value of known same-bird acoustic distance (either within or between years) was 1.0, whereas the lowest acoustic distance between dyads of different individuals was 1.7; consequently no overlap occurred in the acoustic distances of same-bird and different-bird dyads. No significant difference resulted between distances of the same birds recorded in successive years (Laiolo et al. 2007).

In order to identify marked and unmarked individuals recorded from 2004 to 2008, these ‘similarity’ techniques were used on the whole data set of calls (over 4000 vocalizations; Laiolo et al. 2007; Vögeli et al. 2008). Acoustic Euclidean distances between all recorded individuals were calculated, as detailed above. Repeated recordings were considered as belonging to the same individuals when acoustic distances were lower than 1.0, i.e. the acoustic threshold of similarity calculated above.

Overall, we identified 333 individuals with this technique. For this study, we established the life-span (total years from first appearance with a fully developed call to disappearance) of 160 of these 333 males, since we excluded those birds that were solely recorded in the first or last year of study. In doing so, we are confident in capturing the entire life cycle of this short lived-passerine.

**References**

Laiolo, P. & Tella, J.L. (2006) Landscape bioacoustics allows detection of the effects of habitat patchiness on population structure. *Ecology* **87**, 1203-1214.

Laiolo, P., Vögeli, M., Serrano, D., & Tella, J.L. (2007) Testing acoustic versus physical marking: two complementary methods for individual-based monitoring of elusive species. *Journal of Avian Biology* **38**, 672-681.

Laiolo, P., Vögeli, M., Serrano, D., & Tella, J.L. (2008) Song diversity predicts the viability of fragmented bird populations. *PLoS-ONE* **3**, e1822

Specht, R. (2003) Avisoft-SASLabPro. Sound Analysis and Synthesis Laboratory, version 4.23e. Avisoft Bioacoustics, Berlin (Germany).

Vögeli, M., Laiolo, P., Serrano, D., & Tella, J.L. (2008) Who are we sampling? Apparent survival differs between methods in a secretive species. *Oikos* **117**, 1816-1823.
