# Supplementary material for: Multilevel Selection and Neighbourhood Effects from Individual to Metapopulation in a Wild Passerine
Source: PLoS One. 2012 Jun 20;7(6):e38526. doi: 10.1371/journal.pone.0038526 (PMC3380010; doi:10.1371/journal.pone.0038526)
Supplement: Appendix S4 — Group fitness components: population viability. (DOC) [file pone.0038526.s004.doc]

**Appendix S4. Group fitness components: population viability**

The annual rate of population change λ was obtained by means of population viability analyses (PVA), as detailed in Laiolo et al. (2008) and Vögeli et al. (2011). In short, simulations of the stochastic and deterministic forces affecting the Ebro Valley metapopulation were run 100 times by means of the Program VORTEX (Miller & Lacy 2005); the time frame was set at 100 years and extinction was defined as only one sex remaining in the population. Life-history parameters entered in simulations refer to dispersal among populations (10% of dispersing individuals among populations < 15 km apart), reproductive rates (maximum productivity per female=5.52, calculated as the double of published average clutch size (2.76), since two clutches per year are laid); mortality rates (as obtained from capture-recapture survival models performed on the data set of birds marked acoustically; a value of φ = 0.46 resulting from survival analyses was entered in PVA); initial population size (as derived from territory mapping technique), carrying capacity (the maximum density observed in a population multiplied by patch size), and annual breeding performance. The latter was assessed by using acoustic evidence, since yearling calls can be easily distinguished from those of adults in the early learning phase [see Laiolo et al. (2008) for spectrographic evidence]. Territorial calls are given by lark yearlings since their first summer while attempting to recruit; being the first vocalization uttered (the song is given in the successive spring), it includes amorphous syllables whose quality improves gradually in a process that lasts less than 30 days in the short recruitment period. By quantifying the relative proportion of yearlings calling in the whole population of vocalizing males in the summer-autumn period, an estimate of yearling-to-adult male ratio was obtained and used as a surrogate of population productivity [see Laiolo et al. (2008) and Vögeli et al. (2011) for discussion on method reliability]. Yearlings in the early learning phase were excluded from estimates of life-span, which only involved birds giving the definitive calls (Appendix 2).

We ran very conservative simulations, supposing that no catastrophes or inbreeding depression occurred. We built different scenarios with respect to the percentage of successful breeding females, in order to incorporate in simulations a correction for nesting failure, which is quite high and due to predation of ground-nesting passerines of Spanish steppelands (Yanes & Suarez 1996). Percentages of successful breeding females were set at 100, 90, 80, 70, 60, 50, and 40%; λ was calculated by averaging the seven values from the different simulation scenarios.

**References**

Laiolo, P., Vögeli, M., Serrano, D., & Tella, J.L. (2008) Song diversity predicts the viability of fragmented bird populations. *PLoS-ONE,* **3**, e1822

Miller, P.S. & Lacy, R.C. (2005) VORTEX: a Stochastic Simulation of the Extinction Process, Version 9.50.

Vögeli, M., Laiolo, P., Serrano, D., & Tella, J.L. (2011) Predation of experimental nests is linked to local population dynamics in a fragmented bird population. *Biology Letters*, **7**, 954-957.

Yanes, M. & Suarez, F. (1996) Incidental nest predation and lark conservation in an Iberian semiarid shrubsteppe. *Conservation Biology,* **10**, 881-887.
