# Supplementary material for: Multilevel Selection and Neighbourhood Effects from Individual to Metapopulation in a Wild Passerine
Source: PLoS One. 2012 Jun 20;7(6):e38526. doi: 10.1371/journal.pone.0038526 (PMC3380010; doi:10.1371/journal.pone.0038526)
Supplement: Appendix S5 — Contextual analysis models and parameters. (DOC) [file pone.0038526.s005.doc]

**Appendix S5**. Contextual analysis models and parameters.

**Multilevel selection models:**

**Directional selection: ω_ji_= α + β_I_z_j_ + β_G_z_ji_ + ε**

**Disruptive/ Stabilizing selection: ω_ji_= α + β_I_z_i_ + ½ γ_I_z_i_^2^+ β_G_z_ji_ + ½ γ_G_z_ji_^2^+ ε**

**Level 1: Individual and population level analyses**:

ω_ji_ = relative life-span of the focal individual *j* in the *i*th population

α =intercept

z_j_ =repertoire size of the individual *j*

z_ji_ =average repertoire size of the *i*th population

β_I_ = partial regression coefficient of ω_ji_ on z_j_, controlling for population repertoire size (individual selection coefficient)

β_G_ = partial regression coefficient of ω_ji_ on z_ji_, controlling for individual repertoire size (population selection coefficient)

γ_I_ = quadratic selection coefficient for individual repertoire size

γ_G_ =quadratic selection coefficient for population repertoire size

ε =error term

**Level 2: Metapopulation level analyses**:

ω_ji_ = relative annual rate of population change of the focal population *j* with respect to the average rate of population change of the rest of the populations

α =intercept

z_j_ =mean repertoire size of the population *j*

z_ji_ =average repertoire size of the nearest neighbour population

β_I_ = partial regression coefficient of ω_ji_ on z_j_, controlling for mean neighbour-population repertoire size (population selection coefficient)

β_G_ = the partial regression coefficient of ω_ji_ on z_ji_, controlling for mean population repertoire size (metapopulation selection coefficient)

γ_I_ = quadratic selection coefficient for mean population repertoire size

γ_G_ = quadratic selection coefficient for mean neighbour–population repertoire size

ε = error term
