# Supplementary material for: Multilevel Selection and Neighbourhood Effects from Individual to Metapopulation in a Wild Passerine
Source: PLoS One. 2012 Jun 20;7(6):e38526. doi: 10.1371/journal.pone.0038526 (PMC3380010; doi:10.1371/journal.pone.0038526)
Supplement: Appendix S6 — Relationship between relative life-span and individual repertoire size as observed in 32 Dupont’s lark males. Individuals are classified as belonging to groups with average, large (> average+1SE) and small (< average−1SE) repertoires. Trend lines are also shown. (DOC) [file pone.0038526.s006.doc]

**Appendix S6.** Relationship between relative life-span and individual repertoire size as observed in 32 Dupont’s lark males. Individuals are classified as belonging to groups with average, large (> average+1SE) and small (< average-1SE) repertoires. Trend lines are also shown.
