# Supplementary material for: Multilevel Selection and Neighbourhood Effects from Individual to Metapopulation in a Wild Passerine
Source: PLoS One. 2012 Jun 20;7(6):e38526. doi: 10.1371/journal.pone.0038526 (PMC3380010; doi:10.1371/journal.pone.0038526)
Supplement: Appendix S7 — Relative life-span of Dupont’s lark males as predicted by their repertoire size and that of their group mates by the multiple regression model. Multiple regression coefficients obtained from 32 birds of known life-span were fitted to the songs of 155 males from 19 groups. (DOC) [file pone.0038526.s007.doc]

**Appendix S7**. Relative life-span of Dupont’s lark males as predicted by their repertoire size and that of their group mates by the multiple regression model. Multiple regression coefficients obtained from 32 birds of known life-span were fitted to the songs of 155 males from 19 groups.
