# Supplementary material for: Multilevel Selection and Neighbourhood Effects from Individual to Metapopulation in a Wild Passerine
Source: PLoS One. 2012 Jun 20;7(6):e38526. doi: 10.1371/journal.pone.0038526 (PMC3380010; doi:10.1371/journal.pone.0038526)
Supplement: Appendix S8 — Relationship between relative population viability (λ) and the mean song repertoire size of the local population (A) and of the nearest population (B) as observed in 19 populations of the Dupont’s lark. Residuals of the regression of λ on mean population repertoire were used instead of raw values in plot B. (DOC) [file pone.0038526.s008.doc]

**Appendix S8**. Relationship between relative population viability (λ) and the mean song repertoire size of the local population (A) and of the nearest population (B) as observed in 19 populations of the Dupont’s lark. Residuals of the regression of λ on mean population repertoire were used instead of raw values in plot B.
